# Changes in the activity levels and financing sources of Israel’s private for-profit hospitals in the wake of reforms to the public-private divide

**DOI:** 10.1186/s13584-021-00455-z

**Published:** 2021-03-15

**Authors:** Royi Barnea, Adi Niv-Yagoda, Yossi Weiss

**Affiliations:** 1 Assuta Health Services Research Institute, Assuta Medical Centers, Tel-Aviv, Israel; 2 Sackler Faculty of Medicine at Tel Aviv University, Netanya, Israel; 3 School of Health Systems Management at Netanya Academic College, Netanya, Israel; 4 Ariel University, Netanya, Israel

**Keywords:** Health policy, Health economics, Private healthcare system, Public healthcare system, Healthcare funding, Health regulations

## Abstract

**Background:**

The Israeli National Health Insurance Law provides permanent residents with a basket of healthcare services through non-profit public health insurance plans, independently of the individual’s ability to pay. Since 2015, several reforms and programs have been initiated that were aimed at reinforcing public healthcare and redressing negative aspects of the health system, and specifically the constant rise in private health expenditure. These include the “From Reimbursement-to-Networks Arrangement”, the “Cooling-off Period” program and the program to shorten waiting times. The objectives of this study were to identify, describe, and analyze changes in private hospitals in 1) the volume of publicly and privately funded elective surgical procedures; and 2) private health expenditure on surgical procedures.

**Methods:**

Data on the volume and funding of surgical procedures during 2013–2018 were obtained from Assuta Medical Center, Hertzelia Medical Center, the Israeli Ministry of Health and the Central Bureau of Statistics. The changes in the volume and financing sources of surgical activities in private hospitals, in the wake of the reforms were analyzed using aggregate descriptive statistics.

**Results:**

Between 2013 and 2018 the volume of surgical activities in private for-profit hospitals increased by 7%. Between 2013 and 2017, the distribution of financing sources of surgical procedures in private hospitals remained stable, with most surgical procedures (75–77%) financed by the voluntary health insurance programs of the health plans (HP-VHI). In 2018, following the regulatory reforms, a significant change in the distribution of financing sources was observed: there was a sharp decline in the volume of HP-VHI-funded surgical procedures to 26%.

Concurrently, the share of publicly-funded surgical procedures performed in private hospitals increased to 56% in 2018.,. During the study period, private spending on elective surgical procedures in private hospitals declined by 53% while public funding for them increased by 51%.

**Conclusions and policy implications:**

In the wake of the reforms, there was a substantial shift from private to public financing of elective surgical activity in private hospitals.

Private for-profit hospitals have become important providers of publicly-funded procedures. It is likely that the reforms affected the public-private mix in the financing of elective surgical procedures in those hospitals, but due to the absence of a control group, causality cannot be proven. It is also unclear whether waiting times were shortened. Health reforms must be accompanied by a clear and comprehensive set of indicators for measuring their success.

**Supplementary Information:**

The online version contains supplementary material available at 10.1186/s13584-021-00455-z.

## Introduction

The Israeli National Health Insurance (NHI) Law, which went into effect in 1995, provides permanent residents with a basket of healthcare services through non-profit health plans (HPs), independently of the individual’s ability to pay. The HPs provide health services in HP clinics or through the purchase of services from external suppliers, primarily hospitals, which account for the main expenditures of HPs.

Since 2015, the Government of Israel has introduced numerous health system reforms and programs that aimed to reinforce public healthcare, and whose effect has been the restructuring of the health system. These reforms have introduced fundamental changes through legislation (the Economic Arrangements Law), regulations, guidelines, regulatory directives, and new arrangements designed to redress negative aspects of the health system, and specifically the constant rise in private health expenditure.

Based on the CBS data, between 1995 and 2018, the share of public financing for national health expenses declined from 68.2 to 63.8%. This decrease in public financing was apparently due in part to budgetary erosion and limited investments in the public healthcare system while at the same period of time, an increase in private spending was reported. The growth in health expenditure per capita during this period was approximately 1.7% a year, on average, but only 0.9% per age-adjusted standardized person, when considering changes in healthcare prices relative to the Consumer Price Index and increased needs due to changes in the age structure of the population [1]. Moreover, during this period the share of public financing within health expenditure in Israel was relatively low compared to the OECD average (64% in Israel vs. 74% in OECD countries) [2]. In 2018, the share of national spending on health in Israel was merely 7.4% of the country’s gross domestic product (GDP), compared an average of 8.9% in OECD countries [2, 3].

Concurrent with the decline in the share of public financing, a consistent rise in private expenditure on health has been documented over the years through voluntary health insurance (VHI) purchased either through the health plans - policies known as supplementary insurance (HP-VHI) - or through commercial insurance companies (C-VHI). The share of private expenditure on VHI out of total healthcare expenditure increased from 8.2% in 2010 to 11.1% in 2018; about 75% of this increase is attributed to the purchase of commercial insurance [2].

For our discussion here, it is important to distinguish between two distinct, yet interrelated, facets of every health system: healthcare financing and healthcare delivery. According to Article 3 of the NHI Law, the state assumed the obligation of financing the basic governmentally guaranteed health benefits package, while the delivery of most of these services is the responsibility of the (non-profit) HPs. Health services are delivered either directly by HPs or through various external service providers that receive most of their funding by selling services to HPs. These service providers may be owned by the government, by a non-profit organization or a private company.

An important example is the issue of elective surgeries. Prior to the 2015 reforms, elective surgical procedures performed in private for-profit hospitals were covered by a mix of commercial insurance, supplemental insurance, and out-of-pocket payments. Patients could choose the surgeon, the anesthesiologist and even the medical equipment to be used. HPs did not purchase elective operations (a service that is included in the NHI healthcare services basket) from for-profit hospitals. Additionally, the vast majority of public hospitals were not allowed to provide privately financed services that are included in the healthcare services basket; the hospitals located in Jerusalem are an exception for historical reasons. Consequently, at that time, concerns were raised that public hospitals would lose patients and staff to private hospitals due to growing patient interest in securing the benefits of privately-financed care.

The blurring of the lines between health services provider and funding and between private and public health services in Israel is reflected in public and private/semi-private medical services (“SHARAP”, an acronym in Hebrew which stands for “private medical services”) provided by private not-for-profit hospitals in which patients can choose their physician by paying an additional fee. Throughout time, the private-public mix in Israel was the possible origin for concerns that patients receiving care through the public system are deprioritized in terms of access, waiting times, and seniority of the attending specialist in comparison to patients who received the same services but paid for the service through the private system [4, 5].

In view of the vast changes in the health system in recent years, it has become extremely important to track, measure, and identify the effects of the governmental reforms on the health system from their inception (e.g., increased volume of government-financed surgical procedures, mix of health expenditure financing sources, and reduced private spending on health). It is equally important to examine whether these reforms achieve the desired results as defined in the official goals of the Ministry of Health (MoH) and Ministry of Finance (MoF), or whether additional/different steps are necessary to align outcomes with goals. In the section that follows, we briefly review the three main health reforms that were introduced between 2015 and 2018: the “from reimbursement to networks” arrangement, the “cooling-off period,” and the programs to shorten waiting times. Additional details can be found in the appendix.

### Recent health reforms in Israel related to the public-private mix

#### From Reimbursement-to-Networks Arrangement

##### The background to the reform

For many years HP-VHI and C-VHI plans offered their members two main tracks for surgical procedures and medical consultations, allowing members to choose between a monetary reimbursement track and a network-limited benefits track [6]. This kind of insurance was similar to the United States’ Point-Of-Service insurance, which operates as a health maintenance organization for in-network, and as indemnity insurance out of network. All HP-VHI and C-VHI plans offered both tracks, with varying degrees of emphasis on the two. For example, while the corporate and operating structure of Clalit Health Services emphasized the network track, Maccabi Healthcare Services’ operating structure and marketing strategy channeled most of the members of its HP-VHI plans to the reimbursement track. Each track had unique features and effects on the public health system:

##### The financial reimbursement track

In this track, individuals could receive reimbursement for the cost of surgical fees and medical consultations performed by physicians who were not part of the insurer’s network [7]. In general, the reimbursement track effectively gave individuals almost unlimited freedom in choosing a surgeon or specialist for a consultation, including senior physicians who set high, rigid fees and were not affiliated with insurer networks out of financial considerations. This freedom of choice accounted for the marketing power of these plans. However, the clear advantage of freedom of choice was offset by having to pay a relatively high fee.

The reimbursement track was less cost beneficial because the ability of HP members to choose any physician they desire and to receive reimbursement for their expenses created a negative incentive for physicians to join insurers’ networks. In addition, HPs had very little or no control over their spending on this track. Consequently, VHI expenditures on surgical procedures and consultations soared, especially in HP-VHI plans whose reimbursement track was an integral part of their organizational concept.

**(A) The network track**

In this track, when elective procedures and consultations are performed by a physician affiliated with an HP-VHI or C-VHI, the insured individual is charged a co-pay, which may vary from one C-VHI to another and from one VHI plan to another under the same HP. Co-pays are determined according to a range of parameters, for example, the type of surgery, its location (i.e., specific medical center) and the surgeon. In the past, HP-VHI plans could offer coverage with no co-pay for surgical procedures performed in private facilities; however, following the government’s decision within the Economic Arrangements Law of 2008, it was determined that VHI plans would not include coverage of choice of surgeon with no co-pay [6]. This decision was based on the understanding that co-pays potentially restrain demand and effectively counterbalance public indifference to excess use of private health insurance.

### The essence of the reform

In response to the recommendations of the “Committee for Strengthening the Public Health System”, headed by then Minister of Health, Yael German [7], the MoH and MoF resolved to promote a transition from the financial reimbursement method to a network-based system of health services, through the Health Chapter in the Economic Program Law [8]. The stated objective was to reduce private health expenditure. The advantage of the HMOs’ sizes were leveraged to negotiate physicians’ salaries for consulting or performing procedures.

On November 8, 2015, the Knesset Finance Committee approved the regulatory rule that prohibits HP-VHI and C-VHI plans from offering reimbursement for a surgical procedure or medical consultation. Instead, the insured individual must select the service provider from the insurer’s network and will be charged no fee other than a co-pay.

Of note is that concurrently with the changes above, another regulatory rule prohibited insurers and physicians from entering agreements with senior physicians and specialists in unique fields that inevitably drive increased fees for these physicians. Additionally, under this regulatory arrangement there is a limit to the number of services each insured individual can obtain per calendar year.

The Health Chapter of the Economic Program Law, including the transition from the reimbursement track to the network track, went into effect on July 1, 2016 despite harsh criticism voiced mainly by the Israel Medical Association.

The transition to the network track was intended to improve HPs’ control over their expenses, especially for those HPs that had a high percentage of members who chose the reimbursement track.

By promoting competition among HPs over the size and quality of their networks, the reform sought to achieve the NHI Law’s fundamental aim to promote competition among HPs over accessibility, availability, and service quality.

**B. The “cooling-off period” regulations.**

Concurrently with the transition from reimbursement to network arrangement described above, the Public Health Regulations were amended (effective November 2017) to include a “cooling-off period” [9]. These regulation stipulated that a physician who treated a patient in the public health system (either in a hospital or in the community) may not treat or give a consultation to that patient privately until 6 months have elapsed (i.e., the cooling-off period). Notably, the cooling-off regulations do not apply to treatments and consultations related to (a) child development; (b) in vitro fertilization and early or advanced pregnancy scans, provided that these are not included in the Second Addendum to the NHI Law; and (c) invasive procedures whose frequency of performance in the previous year was lower than 1:40,000 population.

An analysis of the MoF and MoH’s explanatory notes to the legislation indicates that the regulations were designed to create a limited-period barrier between the public and private health systems, especially with respect to financing. The official aim of the separation was to limit physicians’ ability to divert patients from the public to the private system based on the physicians’ financial interests. The separation is also intended to be used as an additional regulatory tool to restrain the increase in private health financing that stemmed from the relatively high co-pays on surgical and elective procedures performed in the private health system (through VHI plans).

From its outset, the “cooling-off period” regulations attracted fierce criticism from the medical profession. In a petition filed with the High Court of Justice [10], the petitioners argued that these regulations would potentially harm physicians and the public health system, negatively effecting the public due to: (a) increased HP deficits because of a rise in publicly funded operations and additional procedures in the private system; (b) increased volume of activity in the public health system that will result in long waiting periods, while operations and other procedures will be deferred to later dates to comply with the six-month cooling-off restriction; (c) senior physicians will refrain entirely from working in outpatient clinics of public hospitals to avoid restricting their privately paid procedures; (d) the regulations restrict patients’ choice of surgeons in the private health system.

**C. The program to shorten waiting times.**

The program to shorten waiting times was launched in September 2016 in order to: 1) address the problem of lengthy waiting times for elective procedures in the public health system by increasing the number of procedures (regardless of the identity of the provider - private/public), and 2) reduce private health expenditure in the component of co-payments for surgeries and procedures by allocating additional budget to the healthcare system so that more operations may be performed. Unlike the previous two steps, which were only regulatory rules, the program to shorten waiting times is a budgetary program, that was accompanied by an additional government allocation of NIS 870 million (250 million USD) for direct support to HPs and NIS 180 million to public hospitals (approximately 1.7 and 0.4%, respectively, of the money allocated to the healthcare services basket). The MOH stipulates participation in the program by providing a full report comprising information on the funding body, actions and diagnoses according to ICD9 codes, MOH price list codes, and approval of the completeness of the report [11].

According to the MoH and MoF, the success of the program to shorten waiting times depends, among other things, on the success of corresponding regulatory arrangements, and especially the “cooling-off period” program. In November 2017 criteria for direct public support to HPs were published. These included increased volume of publicly funded operations and other procedures, a decline in activities funded by VHI plans, and criteria for diverting surgical procedures whose fees were under NIS 6000 to public funding. On September 23, 2019, a list of 115 authorized public and private providers in the program was published [12].

Notably, as no specific targets or measures for assessing waiting times were defined, this lacuna naturally affected the regulator’s ability to evaluate the program’s success and the extent to which it achieved its official aim.

In light of all of the legislative changes and regulatory arrangements described above, we aimed to identify, describe, and analyze 1a) the volume of publicly and privately funded elective surgical procedures, 1b) the distribution of financing sources of surgical procedures in the health system, 1c) private health expenditure on surgical procedures and 2) the extent to which the reforms’ aims were met.

## Materials and methods

### Data sources

Data on the volume of elective surgical procedures performed between 2013 and 2018 in private hospitals were obtained from Herzliya Medical Center and Assuta Medical Centers (which operate as private for-profit hospitals across Israel). Data representing all hospitals in Israel (including all types of ownership structures) were obtained from the MoH.

Financial data was obtained from The Summary Report of the Division for Supervision and Control on Health Funds and Additional Health Services [13] and from hospitals’ reports to the MoH. In addition, data on the distribution of payments according to sources of funding were obtained from Herzliya Medical Center and Assuta Medical Centers. These data distinguish between the following sources: public - Form 17, supplementary insurance, private insurance and “out-of-pocket payments”.

### Data analysis

As it was not possible to discriminate among the outcomes of the three reforms, the analysis examined their outcomes together and compared data before and after the reforms. It is notable that the authors performed in-depth interviews with health policy leaders in Israel in order to distinguish between the different reforms. These findings will be elaborated in a future study.

## Results

### The volume of publicly and privately funded elective surgical procedures

**Fig. 1:**
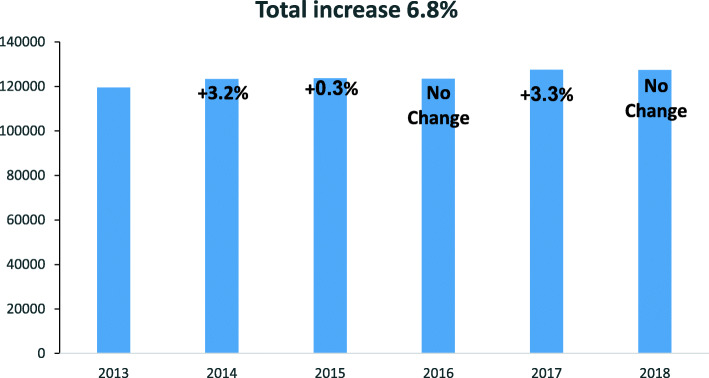
Number of surgical procedures in private hospitals, 2013–2018. Data was obtained from Assuta Medical Centers (nationwide data) and Herzliya Medical Center. Total increase in number of surgical procedures during the analyzed period was 8.6%

Between 2013 and 2018 the volume of surgical activities in private for-profit hospitals increased by 6.8% (Fig. 1).

### Distribution of financing sources of surgical procedures

**Fig. 2:**
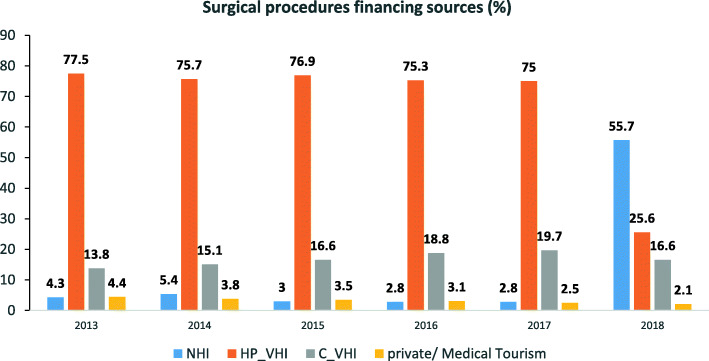
Percent of surgical procedures in private hospitals by year and funding source, 2013–2018. NHI, National Health Insurance Law; HP-VHI, health plan boluntary health insurance; C-VHI, commercial voluntary health insurance

Between 2013 and 2017 the distribution of financing sources of elective surgical procedures in private hospitals was fairly stable. In that period, most surgical procedures (75–77%) were financed by HP-VHI plans. There was a gradual increase in the share financed by C-VHI plan, from 14 to 20%. The rest was financed by the NHI basic healthcare package, private insurance plans and medical tourism. In 2018, however, after the new regulatory arrangements regarding the public-private mix went into effect and the budget for the program to shorten waiting times was transferred to the health system, there was a significant change in the distribution of financing sources: the volume of publicly funded (i.e., covered by the NHI basic benefits package) surgical procedures performed in private hospitals increased dramatically from a single-digit share in previous years to 56%. Concurrently, there was a sharp decline in the volume of HP-VHI-funded surgical procedures - from > 70% in previous years to 26% in 2018 (Fig. 2). Furthermore, after several years of steady increase in the volume of surgical procedures financed by C-VHI plans, the share of C-VHI-funded procedures slightly declined by 3% in 2018 (Fig. 2).

### Private health expenditure on surgical procedures

**Fig. 3:**
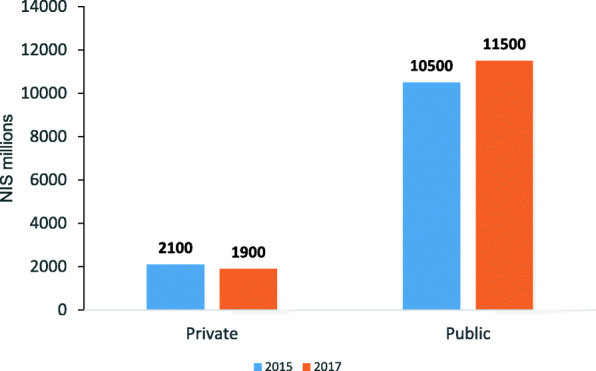
Activity volume (P × Q) of elective surgical procedures, by financing source, 2015–2017. P × Q: activity volume (price*quantity)

After the reforms went into effect there was a 10% decline in private funding and a 10% increase in public funding of elective surgeries in Israeli profit-hospitals (Fig. 3).

## Discussion

Our analysis presents a striking result for the change in the financing source of elective surgeries in for-profit private hospitals following the reforms in the Israeli health system. The findings of the study and data from the Ministry of Health support the study’s hypothesis that following the reforms publicly-funded activities in the private hospitls increased, while there was a certain decrease in private health expenditure (co-payments for surgeries and procedures). Our analysis suggest that the regulatory arrangements, and in particular the program to shorten waiting times, probably strengthened private hospitals’ roles as service providers for HPs. That is reflected in the increased activity of publicly funded healthcare services delivered by private providers. In the wake of the reforms, there was an increase in the production infrastructure of publicly funded procedures and surgeries in parallel to a decrease in funding of procedures and surgeries through the HP-VHI (SHABAN) complementary health service programs.

The allocation of resources for publicly funded surgeries routed surgeries to private providers. Among other things, the reasons for this diversion probably includes these providers’ higher capacity and higher utilization of operating rooms [14] and due to longer operating hours in the private system, which means that more time is available for surgery.

The findings of this study illustrate how the components of the health system function in an integrated manner, whereby each movement affects the existing balance and influences a broad range of indicators both directly and indirectly. Occasionally, the true and significant impact of a change is manifested only months or years later, and in the meantime existing distortions in the system might be sustained, new distortions might be created, or the ability to correct long-established adverse effects might be compromised. For this reason it is extremely important to identify how - in the wake of new arrangements in the health system - key parameters change at various points in time, and to compare achievements to the desired goals and targets.

In Israeli public discourse there are often claims that the public is moving rapidly and excessively toward the use of private medical care and the purchase of private insurance. In contrast, the findings of this study indicate that between 2013 and 2018, the rate of surgical activities in private for-profit hospitals increased by 7% only. This is a reasonable rate of increase, considering that the population’s annual growth rate was approximately 2% during this period and the population is also aging [13]. The observed rate of privately funded surgical activity also reflects the longstanding policy to limit approval for the construction of new operating rooms, in effect limiting the private health system’s production function.

Our findings also suggest that the new regulatory arrangements have affected the distribution of private hospital use by financing source. However, due to the absence of a control group, we were unable to assess whether a causal relationship exists. In addition, due to problems in measuring waiting times in the health system, and the absence of a reliable and relevant database for waiting times, it is not possible to assess whether waiting times decreased, in accord with the program’s official aims. It is even possible that in some cases, waiting times may have increased due to the growth of the eligible group (everyone covered under the NHI and not only HP-VHI members) and the materialization of latent demand (through the removal of economic barriers in the form of high co-pays). Even though publicly funded activities in the health system increased and a certain drop in private spending on health occurred, waiting times were not necessarily shortened.

After many years of structurally and operationally blurred boundaries in HPs between publicly funded and privately funded activities, the “cooling-off period” regulations apparently had a significant effect by creating structurally distinct and separate tracks (public or HP-VHI plans) that allow HPs to identify and prevent diversion of members from the public to the private health system.

These findings and conclusions are also reflected in the financial data published annually by the MoH, which indicate that the HP-VHI plans accumulated a surplus of NIS 189,142 thousand after the new regulatory arrangements and the reforms went into effect in 2016. This surplus led, among other things, to the decision in June 2018 to reduce the monthly premiums for HP-VHI plans.

Similar to our findings, which showed an increase in publicly-funded activity in private hospitals, a similar pattern is presented by an MOH analysis related to all hospitals. According to an MoH report dealing with the government-allocated budget for the program to shorten waiting times - only 35% of the money went to the public hospitals (NIS 362 million) while 65% was transferred through the HPs to private hospitals (NIS 686 million). The MOH report demonstrated an increase in public funding (1.3 billion NIS) and a decrease in private funding (865 million NIS) among all four HPs during 2018 compared to 2015 [13]. According to the report, the rate of HP-VHI (SHABAN)-funded procedures decreased. In addition, the MOH report [13] on the SHABAN indicated a similar pattern (A significant reduction in the expenses of the HP-VHI program of the health funds, especially in the sections of surgeries and consultations). In parallel, private hospital data showed increased activity in public funding (Form 17). These reports complement one another and may deepen the understanding of the changes in the balance between funding sources.

The findings of this study and MoH data suggest that the regulatory arrangements led to an increase in the volume of activities in the publicly-funded health system while private out-of-pocket spending on health (co-pays for surgeries and other procedures) declined. Hence, these regulatory reforms, specifically, the reduced waiting times program, apparently strengthened private for-profit hospitals’ role as service providers to HPs. This expanded the infrastructure used for public health system-funded surgical and other procedures, apparently contributing to a concurrent decline in the use of HP-VHI-funded procedures and in private health spending. Notably, the findings of the study indicate that most of the additional budget was allocated to private for-profit hospitals. Therefore, HPs’ implementation of the new regulatory arrangements apparently led to the referral of a significant volume of members to private hospitals for their public health system-funded procedures.

To what extent did this increase in publicly-funded operations in private hospitals coincide with the objectives of the reforms? The answer to this question is not simple, as the reform had multiple objectives. One objective was to decrease private financing of operations, and that was achieved. But it is unclear whether other objectives were obtained: there are no evidence for shroter wating period for elective surgeries. Another objective was to empower and strenghn the public healthcare system and this goal was only partly achieved.

This finding speaks to the criticism voiced by the directors of public hospitals and MoH executives in the government hospital division, who argued against the unfair competition between public and private hospitals, and that public funds are being diverted to private hospitals by HPs.

Moreover, our analysis distinguishes between supply (ownership) and financing of health services. Instead of being excluded, the private system should work together and alongside the public system in order to release bottlenecks, shorten waiting times and thus improve the quality of care. Reducing private funding can serve as a tool to reduce disparities and inequalities in healthcare, weakening the link between the ability to pay and the receipt of health services and leading to a fairer distribution of the system’s limited resources. Continuing the trend of reducing private spending will help make health services accessible to all and will contribute to health equality.

### Limitations

The data presented in the study is limited to Assuta Medical Centers and Herzliya Medical Center, and did not include data for SHARAP services (privately-funded services in non-profit private hospitals that serve the public system) which are public and private medical/semi-private services in which patients can choose their physician in a public hospital by paying an additional fee. However, Assuta Medical Centers and Herzliya Medical Center are the two largest private providers of surgeries and procedures in Israel. It is likely that the corporate connection between these hospitals and the HPs has helped to increase the activity of these hospitals.^1^

It should be noted that the extent to which the changes can be attributed to the reforms described above is limited due to other changes that took place in the Israeli healthcare system during the study period, and the absence of a control group. Since all changes occurred at the same time, it is hard to differentiate among the relative contribution of each reform.

It is notable that our findings, indicating a decrease in private funding and increase in public funding on surgeries are in correlation with the central bureau of statistics, which demonstrated an overall decrease in private healthcare expenditure and an increase in public funding [15].

## Conclusions

The findings of the current study indicate that private for-profit hospitals have become important providers of publicly-funded procedures. The reforms strongly affected the distribution of health expenditure by financing source, but it is unclear if waiting times were shortened.

## Supplementary Information

**Additional file 1.**

## Data Availability

The datasets used and/or analyzed during the current study are available from the corresponding author on reasonable request.

## Footnotes

1 In 2012, the State Comptroller has indicated the private hospitals that perform these main activities: “The largest private hospitals are: Assuta Medical Centers Ltd., Herzliya Medical Center and Elisha in Haifa” According to the Ministry of Health’s data, in 2010 the HPs purchased services worth NIS 613 million from private hospitals such as Assuta, NIS 86 million from Herzliya Medical Center and NIS 65 million from Elisha; therefore data from the two hospitals in the study present a good and reliable indication of system activity.
