# Supplementary material for: Changes in the activity levels and financing sources of Israel’s private for-profit hospitals in the wake of reforms to the public-private divide
Source: Isr J Health Policy Res. 2021 Mar 15;10:23. doi: 10.1186/s13584-021-00455-z (PMC7958701; doi:10.1186/s13584-021-00455-z)
Supplement: Supplementary file 1 — Additional file 1. [file 13584_2021_455_MOESM1_ESM.docx]

**Appenxdinx: Recent health reforms in Israel related to the public-private mix**

1. **From Reimbursement-to-Networks Arrangement**

The background to the reform

For many years HP-VHI and C-VHI plans offered their members two main tracks for surgical procedures and medical consultations, allowing members to choose between a monetary reimbursement track and a network-limited benefits track [6]. This kind of insurance was similar to the United States’ Point-Of-Service insurance, which operates as a health maintenance organization for in-network, and as indemnity insurance out of network. All HP-VHI and C-VHI plans offered both tracks, with varying degrees of emphasis on the two. For example, while the corporate and operating structure of Clalit Health Services emphasized the network track, Maccabi Healthcare Services’ operating structure and marketing strategy channeled most of the members of its HP-VHI plans to the reimbursement track. Each track had unique features and effects on the public health system:

1. **The financial reimbursement track**: In this track, individuals could receive reimbursement for the cost of surgical fees and medical consultations performed by physicians who were not part of the insurer’s network. Maximum reimbursement was based on the terms of the C-VHI or HP-VHI plans, and the insured individual was reimbursed after providing an invoice. For example, in several insurance plans, the insured individual was entitled to be reimbursed for 80% of a consultation fee for up to a maximum of NIS 600 (approximately 170 USD) per consultation [7]. In general, the reimbursement track effectively gave individuals almost unlimited freedom in choosing a surgeon or specialist for a consultation, including senior physicians who set high, rigid fees and were not affiliated with insurer networks out of financial considerations. This freedom of choice accounted for the marketing power of these plans. However, the clear advantage of freedom of choice was offset by having to pay a relatively high fee. Because the insured individual had only limited ability in negotiating with surgeons over their fees, compared with HPs’ ability to selectively negotiate as a collective with powerful purchasing ability, and especially when senior physicians with unique specialties were involved, in some cases the difference between the actual payment to the surgeon and the maximal reimbursement granted by the insurer could reach tens of thousands of NIS.

The reimbursement track was less cost beneficial because the ability of HP members to choose any physician they desire and to receive reimbursement for their expenses created a negative incentive for physicians to join insurers’ networks. In addition, HPs had very little or no control over their spending on this track. Consequently, VHI expenditures on surgical procedures and consultations soared, especially in HP-VHI plans whose reimbursement track was an integral part of their organizational concept. Because VHI plans operated as independent profit centers, as specified under Article 10 of the NHI Law, their accumulating deficits were translated into a consistent rise in insurance premiums, and consequently, to an increase in private spending on health.

1. **The network track**: In this track, when elective procedures and consultations are performed by a physician affiliated with an HP-VHI or C-VHI, the insured individual is charged a co-pay, which may vary from one C-VHI to another and from one VHI plan to another under the same HP. Co-pays are determined according to a range of parameters, for example, the type of surgery, its location (i.e., specific medical center) and the surgeon. In the past, HP-VHI plans could offer coverage with no co-pay for surgical procedures performed in private facilities; however, following the government’s decision within the Economic Arrangements Law of 2008, it was determined that VHI plans would not include coverage of choice of surgeon with no co-pay [6]. This decision was based on the understanding that co-pays potentially restrain demand and effectively counterbalance public indifference to excess use of private health insurance.

The essence of the reform

In response to the recommendations of the "Committee for Strengthening the Public Health System", headed by then Minister of Health, Yael German [7], the MoH and MoF resolved to promote a transition from the financial reimbursement method to a network-based system of health services, through the Health Chapter in the Economic Program Law [8]. The stated objective was to reduce private health expenditure. The advantage of the HMOs’ sizes were leveraged to negotiate physicians’ salaries for consulting or performing procedures.

On November 8, 2015, the Knesset Finance Committee approved the regulatory rule that prohibits HP-VHI and C-VHI plans from offering reimbursement for a surgical procedure or medical consultation. Instead, the insured individual must select the service provider from the insurer’s network and will be charged no fee other than a co-pay.

Of note is that concurrently with the changes above, another regulatory rule prohibited insurers and physicians from entering exclusive agreements with senior physicians and specialists in unique fields that inevitably drive increased fees for these physicians. Additionally, under this regulatory arrangement there is a limit to the number of services each insured individual can obtain per calendar year. This regulation/arrangement also includes the following exception: HP-VHIs may provide lists of up to 50 physicians who are specialists in unique fields, and whose services will remain subject to the reimbursement track. This exception was designed to allow HPs the flexibility of compensating patients for the services of a small group of senior specialists who might refuse to join their networks.

The Health Chapter of the Economic Program Law, including the transition from the reimbursement track to the network track, went into effect on July 1, 2016 despite harsh criticism voiced mainly by the Israel Medical Association.

The transition to the network track was intended to improve HPs’ control over their expenses, especially for those HPs that had a high percentage of members who chose the reimbursement track. Furthermore, the network track could allow insurers to identify and distinguish among the various expenditures (hospitals, surgeons’ fees, medical devices, etc.), enabling more efficient and effective expenditure management. For example, both HPs and commercial insurers could use their purchasing power to negotiate with physicians concerning inclusion into their respective network lists and significantly reduced the fees charged by the networked physicians

By promoting competition among HPs over the size and quality of their networks, the reform sought to achieve the NHI Law’s fundamental aim to promote competition among HPs over accessibility, availability, and service quality.

**B. The "cooling-off period" regulations**

Concurrently with the transition from reimbursement to network arrangement described above, the Public Health Regulations were amended (effective November 2017) to include a “cooling-off period” [9]. These regulation stipulated that a physician who treated a patient in the public health system (either in a hospital or in the community) may not treat or give a consultation to that patient privately until 6 months have elapsed (i.e., the cooling-off period). Notably, the cooling-off regulations do not apply to treatments and consultations related to (a) child development; (b) in vitro fertilization and early or advanced pregnancy scans, provided that these are not included in the Second Addendum to the NHI Law; and (c) invasive procedures whose frequency of performance in the previous year was lower than 1:40,000 population.

An analysis of the MoF and MoH’s explanatory notes to the legislation indicates that the regulations were designed to create a limited-period barrier between the public and private health systems, especially with respect to financing. The official aim of the separation was to limit physicians’ ability to divert patients from the public to the private system based on the physicians’ financial interests. The separation is also intended to be used as an additional regulatory tool to restrain the increase in private health financing that stemmed from the relatively high co-pays on surgical and elective procedures performed in the private health system (through VHI plans).

From its outset, the "cooling-off period" regulations attracted fierce criticism from the medical profession. In a petition filed with the High Court of Justice [10], the petitioners argued that these regulations would potentially harm physicians and the public health system, negatively effecting the public due to: (a) increased HP deficits because of a rise in publicly funded operations and additional procedures in the private system; (b) increased volume of activity in the public health system that will result in long waiting periods, while operations and other procedures will be deferred to later dates to comply with the six-month cooling-off restriction; (c) senior physicians will refrain entirely from working in outpatient clinics of public hospitals to avoid restricting their privately paid procedures; (d) the regulations restrict patients’ choice of surgeons in the private health system.

**C. The program to shorten waiting times**

The program to shorten waiting times was launched in September 2016 in order to: 1) address the problem of lengthy waiting times for elective procedures in the public health system by increasing the number of procedures (regardless of the identity of the provider - private/public), and 2) reduce private health expenditure in the component of co-payments for surgeries and procedures by allocating additional budget to the healthcare system so that more operations may be performed. Unlike the previous two steps, which were only regulatory rules, the program to shorten waiting times is a budgetary program, that was accompanied by an additional government allocation of NIS 870 million (250 million USD) for direct support to HPs and NIS 180 million to public hospitals (approximately 1.7% and 0.4%, respectively, of the money allocated to the healthcare services basket). The MOH stipulates participation in the program by providing a full report comprising information on the funding body, actions and diagnoses according to ICD9 codes, MOH price list codes, and approval of the completeness of the report [11].

According to the MoH and MoF, the success of the program to shorten waiting times depends, among other things, on the success of corresponding regulatory arrangements, and especially the "cooling-off period" program. In November 2017 criteria for direct public support to HPs were published. These included increased volume of publicly funded operations and other procedures, a decline in activities funded by VHI plans, and criteria for diverting surgical procedures whose fees were under NIS 6,000 to public funding. On September 23, 2019, a list of 115 authorized public and private providers in the program was published [12].

Notably, as no specific targets or measures for assessing waiting times were defined, this lacuna naturally affected the regulator’s ability to evaluate the program’s success and the extent to which it achieved its official aim.

In light of all of the legislative changes and regulatory arrangements described above, we aimed to identify, describe, and analyze 1a) the volume of publicly and privately funded elective surgical procedures, 1b) the distribution of financing sources of surgical procedures in the health system, 1c) private health expenditure on surgical procedures and 2) the extent to which the reforms’ aims were met.

References

6. Knesset Finance Committee, Israel Knesset (Debate November 8, 2015; 26 Heshvan, 5776).

7. Committee for Strengthening the Public Health System Report. . Jerusalem: Israel Ministry of Health; 2014.

8. The Economic Arrangements Law (Legislative Amendments to Achieve Budgetary Goals and the Economic Policy for the Year 2008) 5768-2008. .

9. Rules for Continued Medical Treatment not under Public Financing Following Medical Treatment in a Public Clinic or Community Clinic 5777-2017.

10. High Court of Justice 1393/16 Israel Medical Association vs. Ministry of Health and others.

11. Budget rule no. 31300212.

12. Authorized public and private providers Jerusalem: Ministry of Health; 2019.
